# Identification of a novel *de novo *mutation in the *NIPBL *gene in an Iranian patient with Cornelia de Lange syndrome: A case report

**DOI:** 10.1186/1752-1947-5-242

**Published:** 2011-06-27

**Authors:** Hamid Galehdari, Roya Monajemzadeh, Habibolah Nazem, Gholamreza Mohamadian, Mohammad Pedram

**Affiliations:** 1 Genetics Department, Shahid Chamran University, Ahwaz, Iran; 2 Biochemistry Department, Payamenoor University, Tehran, Iran; 3 Genetic Center, Welfare Organization, Khuzestan, Ahwaz, Iran; 4 Research Center for Hemoglobinopathies and Thalassemia, Ahwaz, Iran

## Abstract

**Background:**

Cornelia de Lange syndrome is characterized by dysmorphic facial features, hirsutism, severe growth and developmental delay. Germline mutations in the *NIPBL *gene with an autosomal dominant pattern and in the *SMC1A *gene with an X-linked pattern have been identified in Cornelia de Lange syndrome.

**Case presentation:**

A two-month-old Iranian boy who showed multiple congenital anomalies was referred to the genetic center of a welfare organization in southwest Iran. He was the second child of a non-consanguineous marriage, born after full term with normal delivery. His birth weight was 3110 g, his length was 46 cm and his head circumference was 30 cm. Both parents were clinically asymptomatic, with no positive history of any deformity in their respective families.

**Conclusions:**

Sequencing of the *NIPBL *gene from our patient revealed a single-base deletion of thymidine in exon 10 (c.516delT). This mutation presumably results in premature termination at codon 526. We did not observe this mutation in the parents of our patient with Cornelia de Lange syndrome. The results presented here enlarge the spectrum of *NIPBL *gene mutations associated with Cornelia de Lange syndrome by identifying a novel *de novo *mutation in an Iranian patient with Cornelia de Lange syndrome and further support the hypothesis that *NIPBL *mutations are disease-causing mutations leading to Cornelia de Lange syndrome.

## Introduction

Cornelia de Lange syndrome (CdLS; http://www.ncbi.nlm.nih.gov/omim/122470), also known as Brachmann-de Lange syndrome, is a clinically and genetically heterogeneous developmental disorder characterized by growth and mental retardation [1,2]. The prevalence of mild and classic CdLS is estimated to be as high as 1.6 to 2.2/100,000 births [3]. Growth retardation is an almost universal finding in patients with CdLS and typically has a pre-natal onset. Mental retardation in patients with CdLS is often severe, resulting in a mean IQ of 53 [1]. Many patients also demonstrate autism-like behavior and self-injurious behavior [4]. No gender-based predilection has been reported, and no differences linked to maternal age or race has been described [3]. The majority of cases are sporadic, and very few familial cases of CdLS have been reported [4]. Pedigree analyses of several families have demonstrated autosomal dominant inheritance with both maternal and paternal transmission [5].

Multiple genes are considered to be responsible for CdLS, all of which are implicated in sister chromatid cohesion [6]. Mutations in the *NIPBL *gene on chromosome 5p13.1 account for approximately 50% of CdLS cases and have been shown to cause both mild and severe forms of the disease [1]. The *NIPBL *gene is 9.5 kbp in length and contains 47 exons that encode two isoforms of 2804 and 2697 amino acids, termed delangin-A and delangin-B, respectively [1]. The human NIPBL proteins share homology with *Drosophila melanogaster *Nipped-B and Scc2 from the budding yeast *Saccharomyces cerevisiae *[6]. The NIPBL protein is directly associated with chromatin while playing a role in the loading of the cohesin complex that mediates sister chromatid cohesion to chromosomes. It also has a dose-dependent gene-regulatory function [7]. Furthermore, mutations in the *SMC1A *gene cause an X-linked form of CdLS [8]. Mutations in the *SMC3 *gene on chromosome 10 have also been reported to cause CdLS [9]. The *SMC1A *and *SMC3 *genes encode the two mitotic cohesion subunits [10]. Cohesin plays a role in sister chromatid cohesion during mitosis and meiosis, in DNA repair and in gene expression [11]. Evidence that cohesin regulates gene expression is accumulating rapidly [12-14] and supports the hypothesis that developmental deficits in patients with CdLS likely arise from changes in cohesin-regulated gene expression that alter transcription in multiple ways [15,16]. Here, we report the first molecular analysis of the *NIPBL *gene in an Iranian newborn baby diagnosed with CdLS.

## Case presentation

A two-month-old Iranian boy with multiple congenital anomalies was referred to the genetic center of a welfare organization in Ahwaz (southwestern Iran).

### Medical history

**Figure 1:**
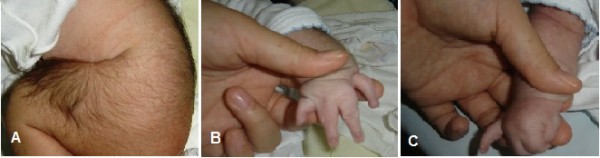
**Characteristic features of Cornelia de Lange syndrome**. (A) Hirsutism. (B,C) Variability of upper-limb abnormalities (distal reduction defect with missing and fused digits in the same child).

At birth, our patient exhibited arch-like confluent (synophrys) eyebrows; long, curly eyelashes; low anterior and posterior hairlines; long philtrum; anteverted nares; depressed nasal bridge; downturned corners of the mouth and thin lips; a high-arched palate; micrognathia; and low-set ears. He also had microcephaly; small hands, with the right hand missing one digit and the left hand having fused digits; hypertonicity; excessive body hair (hirsutism) on the back and feet; cryptorchidism; a small penis; and a low-pitched cry (Figure 1). Echocardiography revealed both an atrial septal defect and a ventricular septal defect in the newborn. Results of kidney sonography were normal. A computed tomography (CT) scan of the brain showed subarachnoid hemorrhage (SAH) and intra-ventricular hemorrhage (IVH) with no sign of hydrocephalus.

### Family history

Our patient was the second child of a non-consanguineous marriage, born after full term by normal delivery, with a birth weight of 3110 g, length of 46 cm and head circumference of 30 cm. Both parents were clinically asymptomatic, with no positive history of any deformity in their respective families.

## Discussion

Three family members (our patient and his parents) were included in this study after informed consent was obtained. Genomic DNA was extracted using a standard protocol, and 46 coding exons (from exons 2 to 47) of the *NIPBL *gene were amplified by polymerase chain reaction assay as described previously [2]. Direct sequencing of the coding exons along with the flanking intron regions of the *NIPBL *gene was performed using the Big Dye Terminator Cycle Sequencing Ready Reaction Kit (Applied Biosystems; Darmstadt, Germany) on an ABI Prism 3700 automated genetic analyzer (Applied Biosystems).

**Figure 2:**
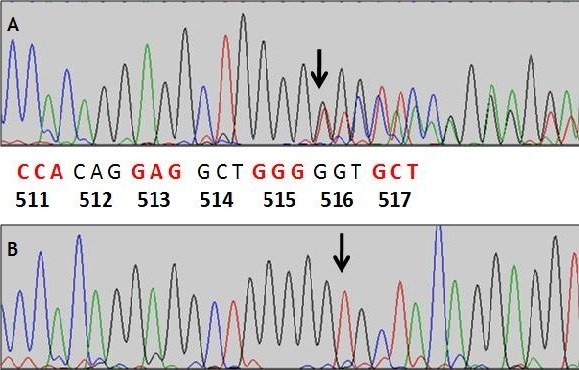
**Sequencing of the *NIPBL *gene**. (A) Chromograph from the affected individual showing a heterozygous deletion of a thymidine (-T) at codon 516 (black arrow). (B) Chromograph from a healthy individual with a wild-type sequence. Letters between both chromographs indicate the partial cDNA sequence of the *NIPBL *gene with corresponding codons beginning with codon 511.

Direct sequencing analysis of the proband demonstrated a heterozygous, single-nucleotide deletion (c.516delT) in exon 10 of the *NIPBL *gene, which resulted in a frame shift, leading to premature termination at codon 526 (Figure 2). Because of the absence of this mutation in the parents of our patient, we report here a novel *de novo *mutation of the *NIPBL *gene. To support the pathogenic nature of this mutation, we searched the genomes of 45 healthy individuals for similar mutations the *NIPBL *gene, with negative results.

## Conclusions

The clinical features of CdLS vary widely among patients, ranging from the classic form, which is severe, to mild forms and including some individuals who have non-syndromic phenotypes but some form of mental retardation [17]. In spite of the differences in severity, the facial dysmorphisms have provided the most helpful features in establishing a diagnosis [4]. In patients with a mild clinical presentation, the characteristic facial appearance may not develop until two to three years of age, while it is always present at birth in the severe form [18]. Structural malformations primarily affect the ulnar aspects of the upper limbs and can range from severe reduction defects, with almost complete absence of the forearms, to small hands with fifth-finger clinodactyly and proximally placed thumbs. Developmental delays and mental retardation are generally moderate to severe. With the aid of molecular analyses, it has recently been recognized that many patients with mild CdLS display primarily mental retardation without substantial structural differences [18].

Numerous studies have indicated that mutations in *NIPBL *cause both mild and severe forms of CdLS [1,2]. These mutations may cause loss-of-function alleles, and the severity of the syndrome generally correlates with the type of mutation [1]. More severe mutations of the *NIPBL *gene, including deletions, cause more severe disease phenotypes than missense mutations [1]. In contrast, mutations in the *SMC3 *and *SMC1A *genes occur in patients with a mild CdLS clinical presentation, including mild facial structural anomalies, no absence or reduction of limbs or digits, no other major structural anomalies or, in some instances, mild to moderate mental retardation with a non-syndromic phenotype [9].

Here, we describe the first molecular genetics diagnosis of an Iranian patient with classic (severe) CdLS. Our patient exhibited characteristic clinical signs of severe CdLS (distinctive facial appearance, limb reduction, microcephaly, short neck and hirsutism). The severity of CdLS in our patient correlates with previous genetic observations in other patients and supports the *NIPBL *mutation described here as the disease-causing mutation in our patient. The single-nucleotide deletion (c.516delT) in exon 10 described here is, according to information available in the Human Genome Mutation Database [19] and, to the best of our knowledge, a novel heterozygous mutation in the *NIPBL *gene. Since both parents of our patient lack this mutation, it strongly suggests that this mutation arose *de novo *in our patient. Studies including more patients with CdLS in Iran are required to assess the prevalence of this and other *NIPBL *mutations in the Iranian population and their importance for CdLS pathogenesis.

## Consent

Written informed consent was obtained from the patient's next-of-kin for publication of this case report and any accompanying images. A copy of the written consent is available for review by the Editor-in-Chief of this journal.

## Competing interests

The authors declare that they have no competing interests.

## Authors' contributions

HG designed the molecular genetic studies and drafted the manuscript. RM carried out the molecular genetic studies and participated in writing the manuscript. GM made the diagnosis. HZ participated in diagnosis and manuscript editing. MP supervised the study. All authors read and approved the final manuscript.
